# Modified lanthanide-doped carbon dots as a novel nanochemosensor for efficient detection of water in toluene and its potential application in lubricant base oils

**DOI:** 10.1007/s00604-023-05659-5

**Published:** 2023-02-18

**Authors:** Christian Chimeno-Trinchet, Maria Emilia Pacheco, Alfonso Fernández-González, Rosana Badía-Laíño

**Affiliations:** 1 Department of Physical and Analytical Chemistry, Faculty of Chemistry, University of Oviedo, Av. Julián Clavería 8, 33006 Oviedo, Spain; 2 Laboratorio de Investigación y Desarrollo de Métodos Analíticos (LIDMA), Facultad de Ciencias Exactas, Universidad Nacional de La Plata, 47 y 115, 1900 La Plata, Argentina

**Keywords:** Carbon dots, Europium, Water determination;, Carbon nanoparticles, Lubricant

## Abstract

A fast and efficient method was developed for obtaining europium(III)-doped surface-modified carbon dots with a hydrophobic coating. This surface functionalization improved the dispersibility of the nanoparticles in non-polar media, as well as modified the accessibility of water molecules to the europium ions. These two features allowed studying the application of doped carbon dots as moisture nanochemosensor, demonstrating high stability over time of both the photoluminescent signal intensity and the stability of the dispersions. The developed nanochemosensor was used to determine water in toluene with a detection limit of 8.5 × 10^−4^ M and a quantification limit of 2.4 × 10^−3^ M. The proposed system matches and even improves other methodologies for water determination in organic solvents; it has a low detection limit and a fast response time (almost instantaneous) and requires neither expensive material nor trained personnel. The results suggest a promising future for the development of a new sensing phase for moisture determination in lubricant base oil.

**Graphical Abstract:**

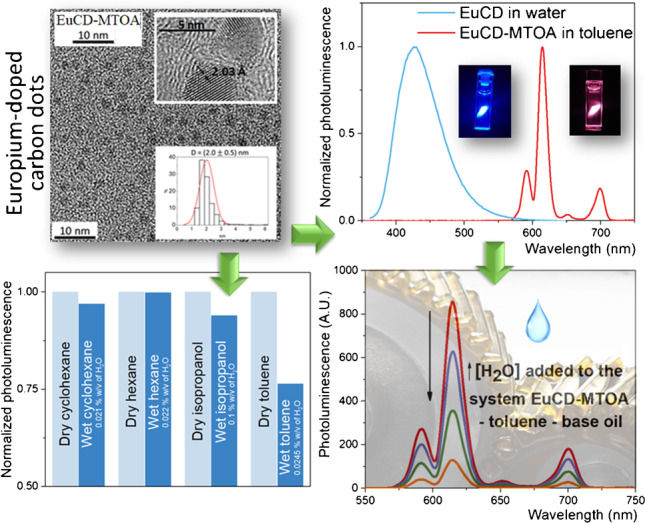

**Supplementary Information:**

The online version contains supplementary material available at 10.1007/s00604-023-05659-5.

## Introduction

Lanthanide photoluminescent complexes have been widely used as fluorescent probes in bioanalytical and technological applications. In particular, Eu(III) complexes offer great advantages due to their narrow emission bands at long wavelengths, large Stokes shifts, and long photoluminescence lifetimes. On the other hand, carbon nanoparticles have attracted great interest among scientists not only due to their intrinsic photoluminescent properties, high photo- and chemostability, high dispersibility, simplicity of synthesis, and possibility of surface functionalization and doping but also due to their biodegradability, reduced cytotoxicity, and low environmental impact [1–3]. Europium and carbon dots (CDs) have already been brought together, studying the interaction between CDs and the complex between europium and tetracycline (TC). The interaction of this type of complex with CDs has also, at the same time, been used to obtain interesting information on stability constants, energy transfer efficiency, and relative distance for the CDs-EuTC association that have helped to delve into the mechanisms that originate the photoluminescent phenomena observed in CDs [3]. Likewise, that knowledge was exploited for determining TC using Eu-doped CDs [4]. Furthermore, the analytical perspective of this combination is clearly established also in the determination of dyes [5, 6] or anthrax biomarkers [7]. However, at present, no hydrophobic europium-doped carbon dots have been reported that take advantage of the change in their photoluminescent properties due to the presence of water for its determination in organic media such as solvents and base oils.

Allowable values of water in oils vary depending on the type of lubricant and its application, but are always related to the concentration limits which affect the lubrication performance or the structural integrity of metal parts. For example, ASTM D4378 “Standard Practice for In-Service Monitoring of Mineral Turbine Oils for Steam, Gas, and Combined Cycle Turbines” determines that a water concentration of 0.1%, 1000 ppm, or 0.056 M is excessive for a turbine system [8, 9]. Water in oils can be present as dissolved, free, and emulsified water. The standard analysis of water in oils can be performed according to ISO 3733, for the determination of free water [10, 11], and DIN 51777–1/-2 which uses the Karl–Fischer method for the determination of total water without differentiating between the forms in which it can be present. Part 1 of the latter standard method describes the direct determination of water in oils, while part 2 refers to the indirect determination [12, 13]. However, one of the main limitations of the Karl Fischer method for the determination of water in oils is the presence of additives since these can interfere, resulting in a higher percentage of water than the actual one [14, 15].

From an analytical point of view, the determination of water in organic media has different approaches, besides the already mentioned Karl–Fischer titration. The literature reveals ratiometric fluorescence sensors [16] and fluorescence probes [17], although the possibility of combining the water sensing probe with interesting tribological properties [18, 19] provides an outstanding added value. In this sense, the europium-doped CDs nanochemosensor proposed in this work can have a double function: as a lubricant additive to improve wear and/or friction and as a water-detecting tool.

In the present work, the synthesis of carbon dots with long lifetimes, obtained by doping with europium salts and surface-modified with ionic liquids to improve their dispersibility in organic solvents, has been addressed. This new material has been successfully used to determine the water content in toluene. Likewise, and as a proof of concept, its suitability as an intrinsic nanochemosensor of water in lubricating oils has been evaluated.

## Experimental

### Reagents and dissolutions

Citric acid monohydrate 99%, L-glutathione > 98% (GSH), methyltrioctylammonium chloride > 97% (MTOA), isopropanol anhydrous 99.5%, toluene anhydrous 99.5%, and Pur-A-Lyzer Mega 1000 dialysis kit were acquired from Sigma-Aldrich; NaOH 98% and europium chloride hydrate were purchased from AlfaAesar; toluene > 99.5%, isopropanol, and hexane > 98.5% were bought to VWR; cyclohexane 99% was purchased from ACROS; and Karl–Fischer reagents were acquired from Honeywell Hydranal.

Water-saturated toluene, cyclohexane, and hexane at room temperature (20 °C) were obtained by contacting 15 mL of the solvent with the same volume of water in a separatory funnel, shaking vigorously for 5 min, and repeating the process 3 times. The organic phase is used once the phases have been separated. For isopropanol, since it is miscible with water, the necessary volumes were mixed to reach the desired w/v%.

Toluene solutions with different water contents were obtained by properly mixing water-saturated toluene and anhydrous toluene. The final molar water concentration in toluene was, therefore, estimated from the solubility of water in toluene (0.0245% w/v, 1.36·10^−2^ M). At 298 K, the solubility of water has been estimated in 0.0098 ± 0.0007% w/w in hexane, 0.008 ± 0.002% w/w in cyclohexane, and 0.0334 ± 0.0003% w/w in toluene [20].

Unadditivated lubricant Base Oil BO68 was kindly provided by REPSOL S.A.

#### Synthesis of europium-doped carbon dots (EuCDs)

The EuCDs were prepared following the solvothermal synthesis previously described by Pacheco et al. [4] (full description in Supplementary information).

#### Hydrophobic modification of EuCDs surface

The surface modification was carried out through an active liquid–liquid extraction of the EuCDs water suspension with toluene as previously described by Chimeno-Trinchet et al. [19] with slight modifications (full description in Supplementary information).

### Instrumentation

Infrared characterization was performed with a Varian 670-IR Fourier transform infrared spectrometer equipped with a golden-gate attenuated total reflectance device. UV–Vis spectrophotometry and photoluminescence experiments were carried out in a Cary 60 UV–Vis spectrometer and a Varian Cary Eclipse spectrofluorometer, respectively. Absolut quantum yields and lifetime experiments were measured in an Edinburgh Instruments FS5 spectrofluorometer. Absolut quantum yields were measured using an integrating sphere. Solutions for quantum yield characterization were used in such a concentration that the absorbance at the absorption maximum was below 0.1. Final quantum yield calculations were performed with the spectrofluorometer software *Fluoracle*.

High-resolution TEM photographs were taken with a JEOL JEM-2100F microscope and an FEI Tecnai F30 microscope. Karl–Fischer experiments were performed in an automatic titrator, Metrohm 899 Coulometer. X-ray photoelectron spectroscopy was carried out in a SPECS spectrometer using monochromatic *K*_α_ Al radiation as excitation source (1486.74 eV) and a flood electron gun for charge compensation.

## Results and discussion

### Morphology and functionalization

**Scheme 1:**
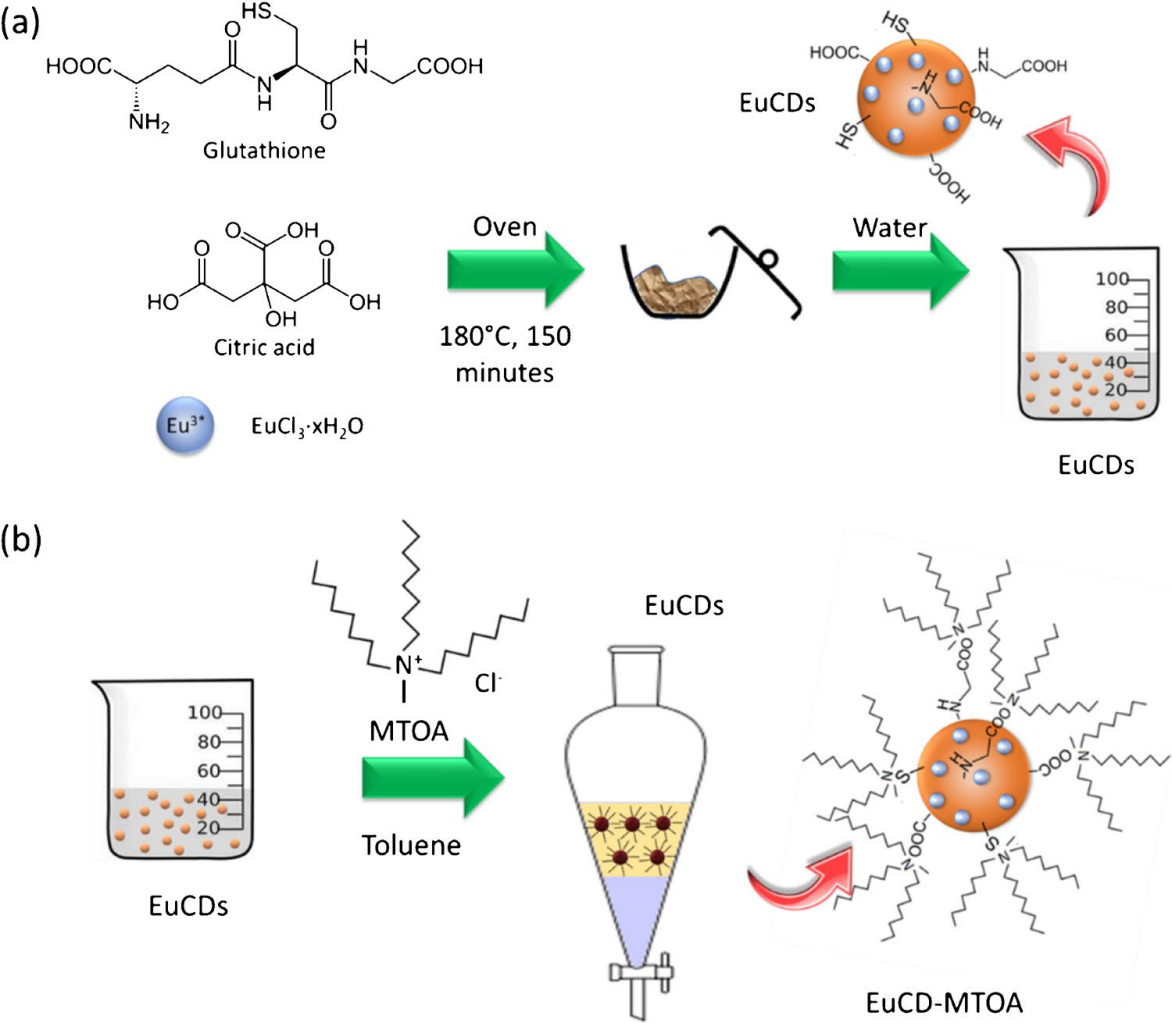
**a** Synthesis of europium-doped carbon dots (EuCD) and **b** hydrophobic modification of EuCDs surface (EuCD-MTOA)

Most of the carbon quantum dot suspensions described in the literature [21] are stable in aqueous solution due to surface functionalization with Lewis acid–base groups mainly derived from the functional groups present in the compounds used as precursors/sources of carbon during its synthesis. Consequently, these CDs have low dispersibility in non-polar media such as certain organic solvents or oils, used in lubrication, food, cosmetics, etc., thus considerably limiting their use in them. Therefore, in order to extend the use of these CDs in non-polar media, the synthesis of the EuCD-MTOA was carried out in two steps, slightly adapting procedures previously described by the authors [4, 19] (Scheme 1): firstly, the doped CDs were synthesized using citrate and glutathione as precursors and europium chloride as a dopant, followed by surface modification using an ionic liquid, methyltrioctylammonium chloride (MTOA). The electrostatic interaction between the hydrophobic cation of the MTOA and the EuCDs allows the extraction and stabilization of the EuCD-MTOA in toluene by the formation of ion pairs, with a structure similar to an inverse micelle [19].

ATR-FTIR was used to compare the different stages of this preparation so as to confirm the hydrophobic surface modification in the EuCD-MTOA. The spectra of both europium-doped and undoped carbon dots (EuCD and CD) do not show significant differences, although these differences are evident when the surface is modified with MTOA. In the latter case, bands at 2990 cm^−1^ and 2845 cm^−1^ were registered, which are typical for alkyl group structures (Figure SI.1) [19].

EuCD and EuCD-MTOA were also characterized using XPS analysis. Due to the inherent characteristics of the C1s high-resolution spectra in complex samples like ours [22], we relayed our interpretation on the comparison of EuCD and EuCD-MTOA spectra (Figure SI.2). The C1s spectrum of EuCD shows a shoulder which is not present in EuCD-MTOA, which is consistent with the hydrophobic coating of the latter. Similarly, the Eu3d signal is also lower in EuCD-MTOA than in EuCD, due to the same reason. A detailed interpretation of the XPS high-resolution spectra is recorded in the Supplementary Material.

EuCD and EuCD-MTOA were morphologically characterized using HRTEM and STEM. The nanoparticles show a monodisperse distribution of spheres between 1.5 and 3 nm with very homogeneous sizes (Figure SI.3). The internal structure of the carbon dots reveals parallel planes with an interplanar distance of 2 Å, indicating a heavy interaction between the planes with a hybridization intermediate between sp2 and sp3. Additionally, STEM-HAADF allows for confirming the presence of europium atoms in the structure of the synthesized carbon dots, corroborated by semiquantitative EDX analysis (0.16 ± 0.04% atomic percentage of europium). A deep discussion of these results is presented in the Supplementary Material.

### UV–vis and photoluminescence spectroscopic characterization

**Fig. 1:**
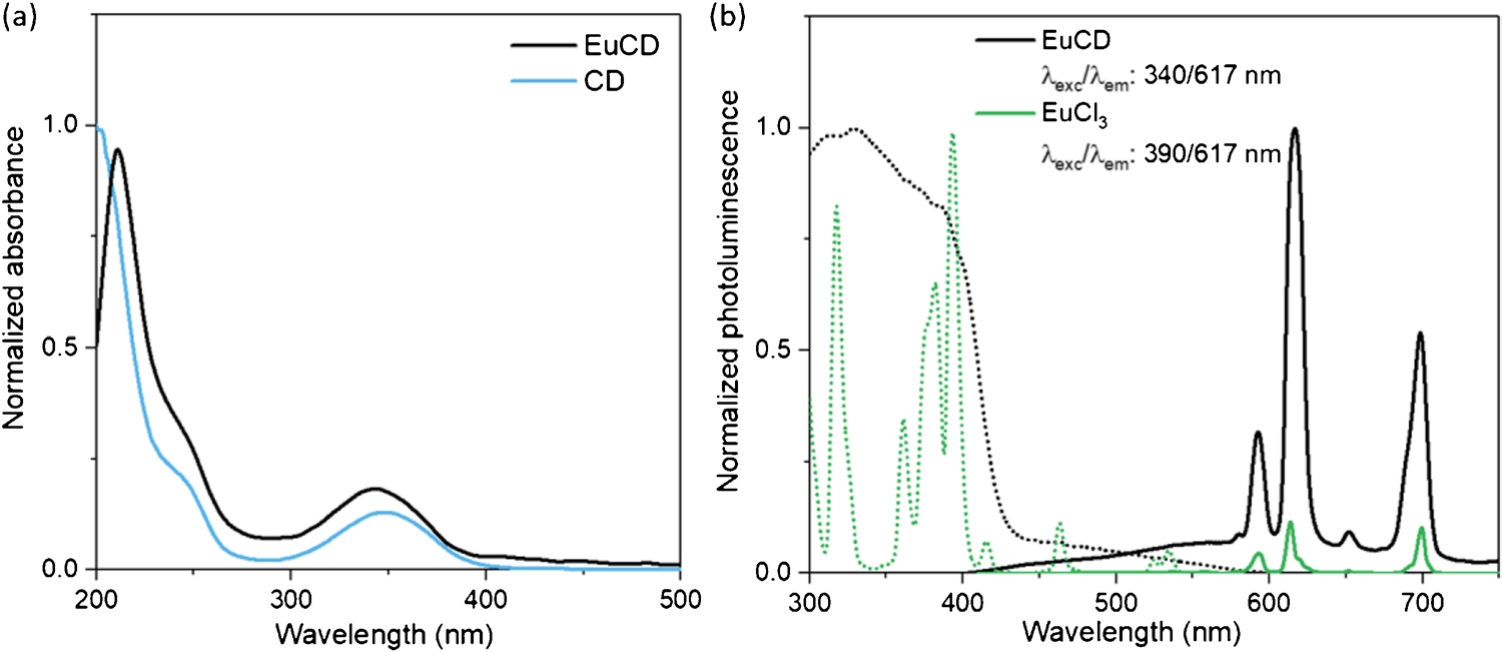
**a** Normalized UV–vis spectra of Eu-doped CDs and CDs suspension in Tris 0.1 M pH 7.4. **b** Normalized photoluminescence spectra of the lyophilized EuCD and EuCl_3_.xH_2_O in FSC (excitation spectra are shown in dotted lines)

The optical properties of the Eu-doped carbon dots were initially studied without surface modification. Figure 1a shows the absorption spectra of 10 ppm suspension of EuCD and CD prepared in Tris 0.1 M (pH 7.4). In both cases, the characteristic bands at 210 and 240 nm belonging to π-π* transitions from C – C bonds with sp^2^ hybridization, as well as a maximum approximately at 350 nm coming from the n-π* transition characteristic of the carbon-heteroatom bond, are observed [23]. This indicates that the presence of the metal ion does not significantly affect the electronic energy distribution in the CDs.

Initially, the photoluminescence studies were carried out in the solid state in order to avoid the solvent effect on the signals. Both the photoluminescence of EuCDs and that of europium salt were measured for the sake of comparison. As shown in Fig. 1b, the photoluminescence spectra for EuCD measured in fluorescence standard conditions (FSC) show an emission spectrum very similar to europium itself with maxima at 594, 614, 617, and 700 nm [3, 4] and an excitation spectrum with a shoulder at 390 nm coinciding with the strongest excitation band of the europium salt. When EuCDs are excited at 340 nm in FSC, corresponding to the excitation maximum of the CD where the europium salt does not exhibit any band, a very low continuous background is revealed and overlaps the clear emission spectrum of the europium ion, despite its low concentration (approximately 1.6% w/w), its low absorptivity coefficient, and the fact that it is rarely directly excited. This fact could be explained by taking into account that the matrix of the carbon dot, where the metal ion is “trapped,” would act as an “antenna” absorbing the radiation and transferring it to the lanthanide; this similarly enhanced environment is observed when chelating compounds are used [24, 25]. The photoluminescence of europium, such as other lanthanides, is due to the electronic transitions in f-orbitals ([Xe]4f^6^) [26] that they are formally forbidden due to the protective effect of the outermost orbitals, and it results in long-lifetime emission like those found in phosphorescence phenomena. The emission spectra of the solids, excited at 340 and 390 nm, measured with a delay time of 0.1 ms and an integration time of 5 ms, and indicated along the text as phosphorescence standard condition (PSC), present a profile identical to that measured under FSC conditions for the europium salt (see Figure SI.4). The fact of performing the measurement under PSC allows monitoring only the europium photoluminescent signal minimizing the intrinsic emission of carbon dots, whose strong emission, when the carbon dots are dispersed in aqueous or organic media, takes place at much shorter times.

**Fig. 2:**
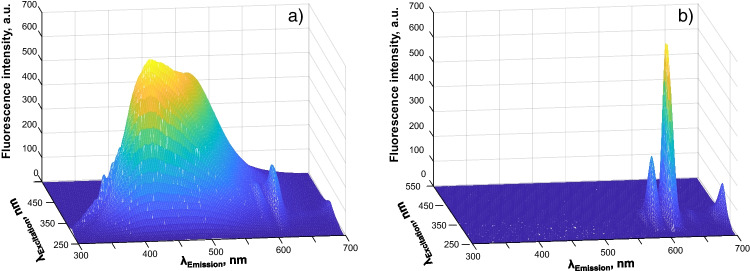
Photoluminescence 3D spectra of EuCD-MTOA dispersed in toluene measured in FSC (**a**) and PSC (**b**)

The photoluminescence properties of EuCD-MTOA in toluene were also fully characterized using both FSC and PSC. When using FSC (Fig. 2a), a strong and wide fluorescence band coming from CD appears. This band is scarcely shifted with the excitation wavelength. Europium emission is also detected in FSC as a poorly intense narrow band at 616 nm in Fig. 2a due to its long lifetime. Notwithstanding, the narrow emission characteristic of atomic photoluminescence of europium is clearly seen when using PSC (Fig. 2b). In this case, the fast photoluminescence belonging to the CDs is already faded and, therefore, not detected.

**Fig. 3:**
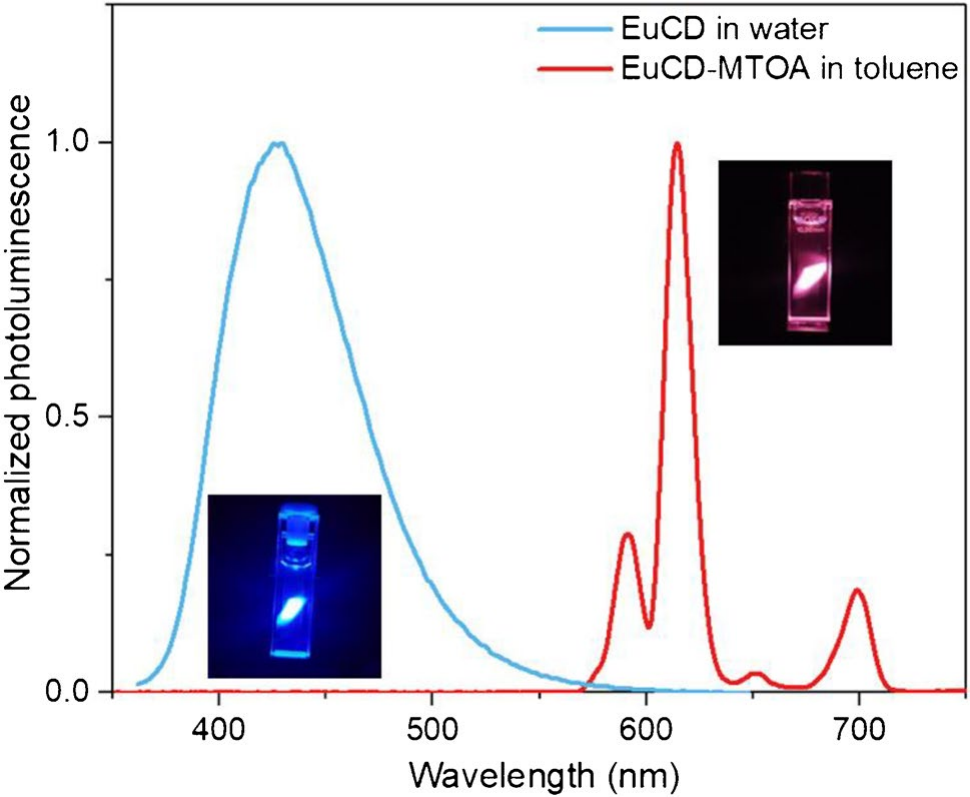
Normalized photoluminescence emission spectra of EuCD in water (FSC) and EuCD-MTOA suspension in toluene (PSC). Excitation wavelength: 340 nm

Further photoluminescent characterizations of EuCD and EuCD-MTOA were performed dispersed in solution. Due to the hydrophobic nature of EuCD-MTOA and the hydrophilic nature of EuCD, the former was suspended in toluene whereas the latter was in TRIS buffer (pH 7.4). A naked-eye inspection of UV-irradiated suspensions shows a blue emission in EuCD and a red emission in EuCD-MTOA (Fig. 3, inlet). The excitation at 340 nm, the excitation maximum of the intrinsic fluorescence of CD regardless of surface modification, produces an intense blue fluorescence emission in both materials at 421 nm when using FSC. However, excitation at 340 nm also generates an intense red photoluminescence emission at 613 nm (typical emission band for europium) when using PSC in EuCD-MTOA toluene suspension, which is not observed in EuCD water suspension (Fig. 3).

This behavior corresponds to the susceptibility of europium emission to the presence of water molecules [27] which, when they enter into the Eu^3+^ coordination sphere, would exert a double effect: as an effective lanthanide photoluminescence quencher due to the presence of non-radiative processes mainly through OH oscillators [25] and favoring the radiative deactivation of the carbon dot. On the contrary, when the carbon dot is in an organic medium, the “antenna” effect dominates, with the attenuation of the CD emission and the enhancement of the europium emission. This effect is also observed in other solvents like n-hexane, cyclohexane, or isopropanol, selected as model aliphatic, cyclic aliphatic, and polar anhydrous organic media, and with increasing polarities [28]. The spectra in PSC at 0.1% w/v suspensions in these anhydrous commercial solvents (with water levels lower than 0.005%) show scarce variations, except for toluene which has a 25% decrease in the signal observed (Figure SI.5). Therefore, the photoemission does not present a clear correlation with the polarity or the KAT parameters and could be considered independent of the nature of the organic solvent (Table SI.1).

**Fig. 4:**
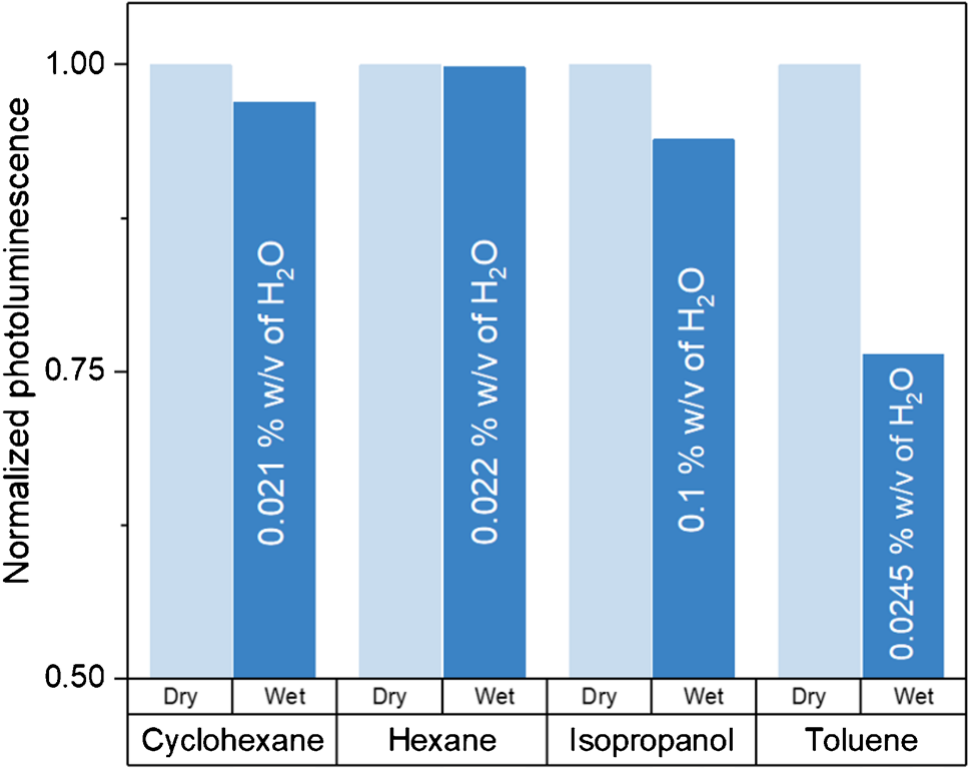
Comparison of EuCD-MTOA (0.1% w/v) photoluminescent emission signals in dry organic solvents (light blue) and solvents saturated with water (dark blue), except for isopropanol that is miscible in water. The water content in each solvent is specified in the figure

Figure 4 shows the photoluminescent emission signals of EuCD-MTOA in PSC collected using commercial anhydrous solvents and compared with those obtained using water-saturated solvents and 0.1% w/v of water in isopropanol (spectra in Figure SI.5). According to the results, toluene is the nonpolar solvent that showed the major differences when comparing anhydrous and saturated solutions. This confirms the sensitivity of EuCD-MTOA to the presence of water, and also, it allows explaining the apparently abnormal results observed for anhydrous toluene in Figure SI.5: its lower photoluminescence emission intensity values, compared to the other anhydrous solvents, can be explained in terms of a higher water content than expected according to commercial specifications, possibly due to water contamination during manipulation.

Additionally, to investigate whether the nature of the organic solvents could affect the electronic band gap of the emission centered at 613 nm, *E*_*g*_^613^, Tauc’s law [29], expressed as a function of the photoluminescence intensity, *P*, was used [3]:

1 $${(Ph\nu)}^2=k'(h\nu-E_g)$$

The minimum energy required to promote an electron from the valence band to the conduction band was calculated from the plot of (Phν)^2^ versus photon energy (hν) (Figure SI.5, inset). The experimental values obtained for *E*_*g*_^613^ were around 2.0 eV for EuCD-MTOA in different media (Table SI.2). Therefore, it follows that the energy of the band gap is not affected by the organic solvent nature, and on the other hand, the effective migration of energy from the CD to the Eu^3+^ would be ascribed to this small gap.

Photoluminescence lifetimes of EuCD in water and EuCD-MTOA in isopropanol, toluene (miscible and non-miscible organic solvent, respectively), and water were determined (Table 1). Decay curves were fitted to the equation *R*(*t*) = Σ_*i*_* α*_*i*_ · *e*^(−*t*/*τi*)^, where *i* was the number of required exponentials (using *χ*^2^ ≤ 1.2 as an indicator of a good fit) and *α*_*i*_ the pre-exponential weighting factor. Also, from the decay data, it was possible to calculate the pre-exponentially weighted mean lifetimes, *τ*_*m*_ [30]. Comparing these last values with those measured with the lyophilized nanoparticles exposed to the air, aqueous suspensions of EuCD show a drastic decrease in their mean lifetimes. This is an expected behavior considering that the lanthanide’s photoluminescence decay times depend on the number of coordinated water molecules [25, 27]. On the contrary, when EuCD-MTOA are suspended in anhydrous organic solvents, high *τ*_*m*_ are obtained: the ionic liquid which renders hydrophobic character to the material act as a protective coating for the europium environment, reinforcing the “antenna” effect of the CD matrix.

**Table 1:** Comparison of photoluminescent properties for EuCD and EuCD-MTOA in different media in PSC

	Φ, quantum yield	*τ*_1_(*α*_1_^a^)	*τ*_2_(*α*_2_^a^)	*τ*_3_(*α*_3_^a^)	*τ*_4_(*α*_4_^a^)	*τ*_*m*_^b^
		Lifetime, µs				
Lyophilized EuCD	0.82%	0.54 (0.826)	106.41 (0.015)	299.79 (0.017)	-	8.31
EuCD water	7.79%	0.09 (0.53)	0.71 (0.01)	9.00 (0)	-	0.10
EuCD-MTOA isopropanol	11.80%	1.14 (1.41)	80.61 (0.03)	518.11 (0.06)	1232 (0.1)	98.95
EuCD-MTOA toluene	8.91%	0.51 (2.1)	79.91 (0.01)	524.18 (0.03)	1280.13 (0.06)	42.91

^a^Pre-exponential weighting factor when photoluminescence decay is fitted to the equation *R*(*t*) = Σ_*i*_* α*_*i*_ · *e*^(*−t*/*τi*)^ (for *χ*2 ≤ 1.2 as an indicator of a good fit)

^b^*τ*_*m*_ = *Σ*_*i*_* α*_*i*_*τ*_*i*_*/Σ*_*i*_* α*_*i*_, 2% of estimated uncertainty for the individual lifetime

### Evaluation of EuCD-MTOA as sensing phase for the determination of humidity in toluene

As previously mentioned, dispersions of EuCD-MTOA in anhydrous and water-saturated toluene showed a marked difference in photoluminescent signals compared to the ones registered for anhydrous and water-saturated dispersions in cyclohexane and hexane and anhydrous and 0.1% w/v water in isopropanol (Fig. 4 and Figure SI.5). This interesting characteristic enables the potential use of EuCD-MTOA as a nanochemosensor for the efficient detection of water in toluene.

In order to study the applicability of EuCD-MTOA as a sensing phase, the stability of the photoluminescent signal of a suspension of EuCD-MTOA (1 ppm) in anhydrous toluene over time was evaluated. The experimental procedure consisted in recording the photoluminescence emission intensity at 613 nm, after excitation at 340 nm (0.1 ms delay time and 5 ms integration time), every 60 min, for a total time of 55 h (3300 min) (Figure SI.6). After that, the dispersion was kept in darkness in a closed vial at room temperature for 2 months, and then, the emission intensity was measured under the same conditions. During the experiment, the signal remained unaltered, with a mean normalized intensity of 0.98 ± 0.02.

Due to the stability of the EuCD-MTOA analytical signal over time, a preliminary study of the influence of water content in the system EuCD-MTOA-toluene, at different concentrations of EuCD-MTOA, was performed. It was observed, as shown in Figure SI.5, that the photoluminescence emission intensity of the EuCD-MTOA-toluene-H_2_O system decreases with increasing water concentration attributable to its character of photoluminescence quencher. The europium ^5^*D*_0_–^7^*F*_2_ transition is easily affected by changes in the coordination sphere of the lanthanide ion; the presence of water molecules favors a non-radiative deactivation pathway that is reflected in the loss of intensity of the emission band at 613 nm. The analysis of the experimental data was performed by means of the Stern–Volmer equation:

2 $${P}_{0}/P = 1 + {K}_{SV} \cdot [Q]$$

where *P*_0_ is the photoluminescence intensity without quencher, *P* is the photoluminescence intensity in the presence of quencher, *K*_*SV*_ is the Stern–Volmer constant, and [*Q*] is the concentration of quencher.

In order to select the optimal working conditions, different concentrations of EuCD-MTOA (0.01, 0.05, and 0.1% w/v) were assayed. Table SI.3 shows the regression curves obtained from the Stern–Volmer plots for the system EuCD-MTOA-toluene when the amount of water added to the system was varied from 0 to 1.36 × 10^−2^ M (this limiting value was due to the formation of a water-toluene emulsion, whose presence would disturb the analytical signal). Analytical sensitivities are also shown.

The statistical evaluation of the slopes (*α* = 0.05) [31] reveals that the one obtained for 0.1% w/v EuCD-MTOA is significantly different from the other two, while there is not a significant difference between the slopes for 0.05% and 0.01% w/v EuCD-MTOA. According to the test, 0.1% w/v EuCD-MTOA would yield better results as the detection capabilities of the system are improved (the major slope is obtained, and it is significantly different from the other two). Besides this fact, the intermediate concentration of EuCD-MTOA was selected in order to increase the number of analyses that can be performed per gram of EuCD-MTOA synthesized, regardless of the lower sensitivity.

**Fig. 5:**
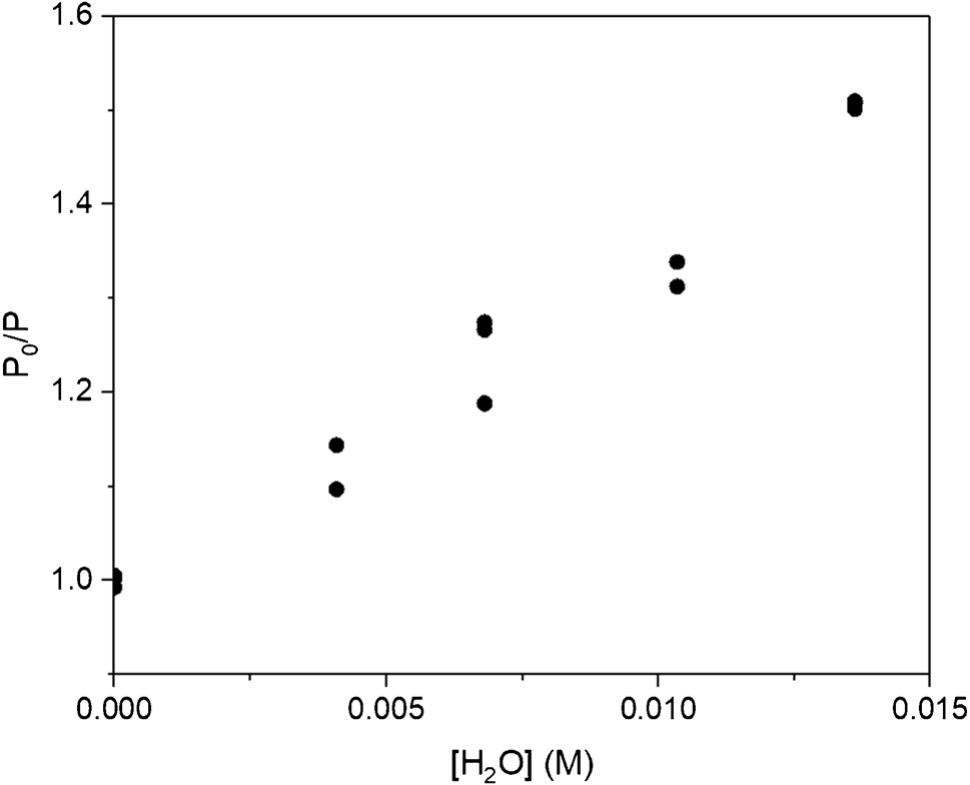
The Stern–Volmer plot for the system EuCD-MTOA-toluene in the presence of increasing amounts of water

In concordance with these preliminary experiments, a calibration curve was performed by means of the Stern–Volmer equation, using 0.05% w/v EuCD-MTOA in anhydrous toluene and varying concentrations of water (0–1.36 × 10^−2^ M). Data points were duplicates at the same concentration level obtained from independent solutions, except for the extremes and middle concentrations where data points were triplicates. As can be seen in Fig. 5, a noticeable dispersion was observed in the replicates of the intermediate concentrations which was probably due to a greater manipulation of suspensions during the preparation, varying the uptake of atmospheric water vapor along the process; this effect was not observed in the extreme points of the curve since those concentrations of water were not obtained from mixtures. The problem of non-constant variance (heteroscedasticity) was solved by using a weighted least-squares procedure [32]. The analytical figures of merit of the proposed methodology were evaluated according to IUPAC recommendations [33], introducing the weighting factors. The precision of the method (expressed as % relative standard deviation) was studied at two concentration levels (3.40 × 10^−3^ M and 1.21 × 10^−2^ M) being replicated three times each on the same day (intra-day precision) and two times each for 3 days (inter-day precision). Optimal experimental/instrumental conditions and the analytical characteristics are shown in Table 2.

**Table 2:** Analytical performance of the developed nanochemosensor for detection and quantification of water in toluene

Optimal conditions	
Experimental	Instrumental
[EuCD-MTOA] = 0.05% w/v	*λ*_exc_ = 340 nm/*λ*_em_ = 613 nm
Solvent: anhydrous toluene	0.1 ms delay time; 5 ms integration time
	Bandwidth: excitation = 7 nm; emission = 3 nm
Analytical figures of merit	
*P*_0_/*P* = (37 ± 1) M^−1^ [H_2_O] (M) + (1.00 ± 0.01); *α* = 0.05; *s*_(*y*/*x*)*w*_ = 0.01	
LOD = 8.5 × 10^−4^ M (0.0015% w/v)	Intra-day precision (*n* = 3): 8.7% RSD (3.40 × 10^−3^ M); 7.9% RSD (1.21 × 10^−2^ M)
LOQ = 2.4 × 10^−3^ M (0.0043% w/v)	Inter-day precision (*n* = 6): 7.9% RSD (3.40 × 10^−3^ M); 5.6% RSD (1.21 × 10^−2^ M)

The performed calibration curve was used for the determination of three toluene samples with different moisture percentages. The Karl–Fisher method revealed a concentration of 5.9 × 10^−3^ M, 8.8 × 10^−3^ M, and 1.2 × 10^−2^ M of water respectively, whereas concentrations of 5.5 × 10^−3^ M, 8.5 × 10^−3^ M, and 1.03 × 10^−2^ M of water were found using the developed nanochemosensor by means of a weighted regression curve. Considering the concentration of the Karl–Fisher method as the expected one, recovery percentages from 84 to 97% were obtained.

### Proof of concept: Preliminary studies for the determination of moisture in base lubricating oils

**Fig. 6:**
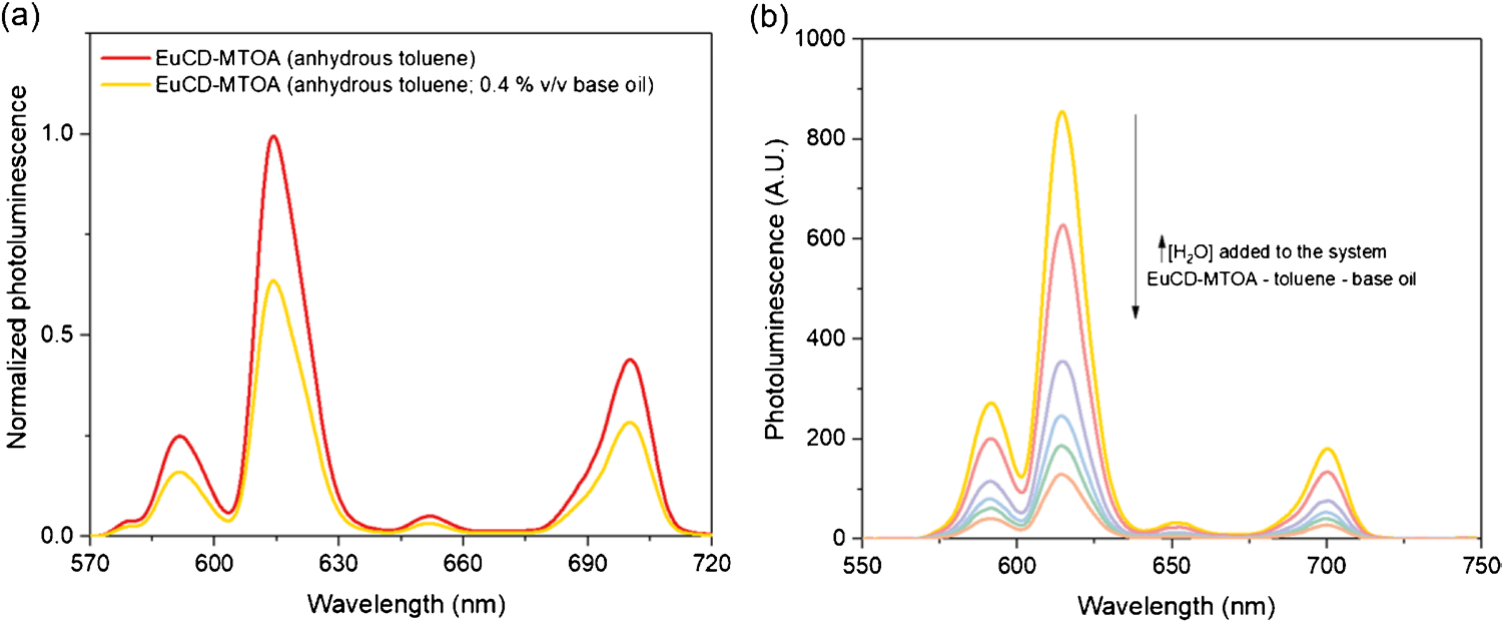
**a** Normalized photoluminescence emission spectra of 0.05% w/v EuCD-MTOA in anhydrous toluene (  ) and in anhydrous toluene and base oil (  ). **b** Photoluminescence emission spectra of the system EuCD-MTOA-toluene-base oil in presence of increasing amounts of water. Measurements taken in PSC

Given the satisfactory results obtained in the evaluation of EuCD-MTOA as a sensor phase for the determination of water in toluene, the potential application of this nanochemosensor in lubricating base oils was assessed. For this purpose, a specified amount of base oil BO68 was added to the suspension of EuCD-MTOA in anhydrous toluene. It was observed that the presence of base oil in the system produces the attenuation of the analytical signal emitted by the EuCD-MTOA at 613 nm by 40% (see Fig. 6a). However, if the amount of fresh BO68 in toluene is kept constant in the system, at a concentration of 0.4% v/v, while varying the percentage of water, there is a linear dependence between *P*_0_/*P* and the amount of water added (M).

These results allow us to anticipate the possibility of developing an adequate methodology for monitoring moisture content in a base oil, and therefore the ageing and contamination. Furthermore, as CD-MTOA have demonstrated intrinsic properties as nanolubricant [19], it is expected that EuCD-MTOA could merge those properties and act as both nanochemosensor and nanolubricant when added to a base oil, expanding their perspectives. Notwithstanding, the potential interference of eventual europium complexants which could be present in lubricants has to be evaluated.

## Conclusions

The synthesized hydrophobic carbon dots doped with europium have shown great potential in the development of new nanochemosensors due to their remarkable sensing properties. The reported methodology has allowed obtaining a new nanoparticle with a high sensitivity to moisture present in non-polar solvents reaching limits very close to other reported methods (Table SI.4). A further study of this sensing phase could lead to the development of sensor kits for fast and reliable moisture determination in environments that do not allow the use of the necessary instrumentation for the Karl–Fisher analysis.

On the other hand, the preliminary studies show that it may be feasible to use the nanochemosensor for the determination of water/humidity in lubricant oils. However, it is necessary to go deeper in the comprehension of the deactivation processes, since they will also depend on the nature of the base oils and, in this way, be able to accurately detect water in real samples.

## Supplementary Information

Below is the link to the electronic supplementary material.

Supplementary file1 (PDF 1258 KB)

## Data Availability

The data supporting the results of this work are available upon a reasoned request to the corresponding author.
