# Supplementary material for: Modified lanthanide-doped carbon dots as a novel nanochemosensor for efficient detection of water in toluene and its potential application in lubricant base oils
Source: Mikrochim Acta. 2023 Feb 18;190(3):97. doi: 10.1007/s00604-023-05659-5 (PMC9938817; doi:10.1007/s00604-023-05659-5)
Supplement: Supplementary file 1 — Supplementary file1 (PDF 1258 KB) [file 604_2023_5659_MOESM1_ESM.pdf]

SUPPLEMENTARY INFORMATION

Modified lanthanide-doped carbon dots as a novel nanochemosensor for efficient detection of water in toluene and its potential application in lubricant base oils

Christian Chimeno-Trinchet[1], Maria Emilia Pacheco[2], Alfonso Fernández-González[1], Rosana Badía-Laíño[1]*.

[1]Department of Physical and Analytical Chemistry. Faculty of Chemistry. University of Oviedo Av. Julián Clavería 8, 33006-Oviedo, Spain

[2]Laboratorio de Investigación y Desarrollo de Métodos Analíticos (LIDMA), Facultad de Ciencias Exactas, Universidad Nacional de La Plata, 47 y 115, 1900 La Plata, Argentina.

*corresponding author: rbadia@uniovi.es; recomol@uniovi.es

Index

1. Synthesis of europium-doped carbon dots (EuCDs)
2. Hydrophobic modification of EuCDs surface
3. XPS Analysis
4. Morphologic characterization
5. Protocol for determination of water in oil
6. Method features compared to other published systems
7. SI Figures
8. SI Tables
9. SI References

1. Synthesis of europium-doped carbon dots (EuCDs)

1 g citric acid plus 0.5 g glutathione and 0.2 g of $EuCl_3 \cdot xH_2O$ were solved into 10 mL ultrapure water and ultra-sonicated until obtaining a homogeneous mixture. The final solution was poured into a porcelain crucible and introduced into an oven at 180 °C for 150 minutes. The mixture in the crucibles was hydrated every 30 minutes (4 times) with

ultrapure water in order to control the carbonization of the reagents. After completing the reaction, the crucibles were removed from the oven and let to cool down in a desiccator, yielding a brownish-orange solid which was dispersed into 15 mL ultrapure water using an ultrasound bath. These final suspensions were purified by dialysis versus water using membranes MWCO 1kDa. Then, the solvent was partially removed using a rotatory evaporator and thereafter lyophilized, obtaining a brown-greenish powder.

2. Hydrophobic modification of EuCDs surface

0.5 g EuCDs were dispersed into 30 mL water and its pH was raised over 10 with NaOH. Meanwhile, 0.3 g (~0.75 mmol) MTOA was dissolved into 30 mL toluene. Both phases were then mixed and vigorously shaken for 72 hours. The organic phase was recovered and washed two times with 30 mL NaOH solution (pH 9 – 10). Toluene was removed with a rotatory evaporator until dry. A brownish viscous almost-solid paste-like material was obtained, EuCD-MTOA.

3. XPS Analysis

According to the work by Gengenbach et al. [22], identifying the organic functionalities in a high resolution C1s spectrum by curve-fitting and assignation of binding energies is poorly trustable and almost speculative when having more heteroatoms than oxygen, as it is our case. Furthermore, the combination of graphitic and non-graphitic carbon which is present in our samples requires different curve profiles and makes the interpretation uncertain. Therefore, we relayed the interpretation on the comparison of EuCD and EuCD-MTOA spectra.

Figure SI.2 (left) shows the high resolution C1s spectra of both EuCD and EuCD-MTOA, revealing that the former has a band at 287.3 eV which is not present in EuCD-MTOA. Such high binding energies are related to highly oxidized carbon states and are sometimes assigned to C=N [34] or to C=O [35], but such assignation is mainly speculative in our case according to Gengenbach's discussion [22]. Nevertheless, we can assume that this band is due to functional groups present in EuCD surface but not in EuCD-MTOA surface, which is consistent with the fact that EuCD-MTOA surface is coated with MTOA (mostly C-C and C-H bonds), which hinders the detection of the underlying functionalities. Similarly, an intense europium peak can be detected for the EuCD sample at 1133.4 eV (Figure SI.2 right), belonging to Eu3d5/2 which fits the position with that of Eu3d5/2 for the inorganic salt $EuCl_3$. This peak is despicable and almost inexistent in

the case of EuCD-MTOA, again because europium is somehow ‘hidden’ below the organic coating of MTOA.

4. Morphologic characterization

EuCD-MTOA were morphologically characterized using HRTEM and STEM. The microphotograph (Figure SI.3a) shows that the suspensions consist of a monodisperse distribution of spherical nanoparticles between 1.5 and 3 nm with very homogeneous sizes. Figure SI.3b also shows the internal structure of the carbon dots, with parallel planes with an interplanar distance of 2 Å. This distance is lower than that of the [002] planes of graphite, indicating a heavy interaction between the planes with a hybridization intermediate between sp^2 and sp^3[36]. Size distribution for EuCD-MTOA and EuCD without hydrophobic layer are also shown in Figure SI.3 d and e.

A microphotograph obtained with STEM-HAADF (Figure SI.3c) allows confirming the presence of europium atoms in the structure of the synthesized carbon dots. The presence of such heavy atoms ($_{63}Eu$) is revealed by a more intense signal than that of the rest of the atoms of the matrix, as the latter show very low atomic numbers and, therefore, a weaker signal [37]. This result was corroborated by semiquantitative EDX analysis which reflected a 0.16 ± 0.04 % atomic percentage of europium.

5. Protocol for determination of water in oil

The proof of concept for moisture determination in lubricating oils was performed as follows: First, a suspension of the EuCD-MTOA was prepared in anhydrous toluene. An appropriate volume of this suspension was added to each flask and dried in a vacuum oven (final concentration of EuCD-MTOA: 0.05 % w/v). After this step, a proper aliquot of the lubricant oil was added to the flask in order to reach a concentration of 10 % v/v. Next, appropriate volumes of anhydrous toluene and water-saturated toluene were added to perform the calibration curve, starting (zero curve point) with anhydrous toluene up to a final concentration of water given by the water-saturated toluene. The solutions were homogenized gently and the whole procedure was carried out quickly to avoid any contamination by environmental humidity.

6. Method features compared to other published systems

The determination of water in organic solvents can be faced from different perspectives. The use of a naphtalimide-based molecule attached to a carbon dot [38] provides a very

sensitive method for determining water in toluene, which is also applicable in lubricant oils, even those with high viscosity. However, that methodology requires a tedious synthesis procedure. Although less sensitive, our proposal uses a fast and simple synthesis which, additionally, produces few residues and is therefore 'greener'.

Similarly, the use of Cs_4PbBr_6 nanocrystals provides a ratiometric fluorescent method for the determination of water in some organic solvents with a similar detection limit to ours and applicability in a wider range of solvents [39]. Nevertheless, since the analytical features are similar, the use of less toxic Carbon Dots should be rather preferred to materials containing lead or toxic metals.

Likewise, there are published descriptions of carbon dots used for the determination of water content in different solvents [40]. For example, carbon dots from 2,5-dihydroxyterephthalic acid can be used for water determination in ethanol and other solvents using a fluorescence ratiometric approach. However, this methodology has a worse limit of detection than the one presented here, as well as a longer response time.

7. SI Figures

Figure SI.1. ATR-FTIR spectra of synthesized carbon dots (CD and EuCD), functionalized CDs (EuCD-MTOA) and $EuCl_3{\cdot}xH_2O$.

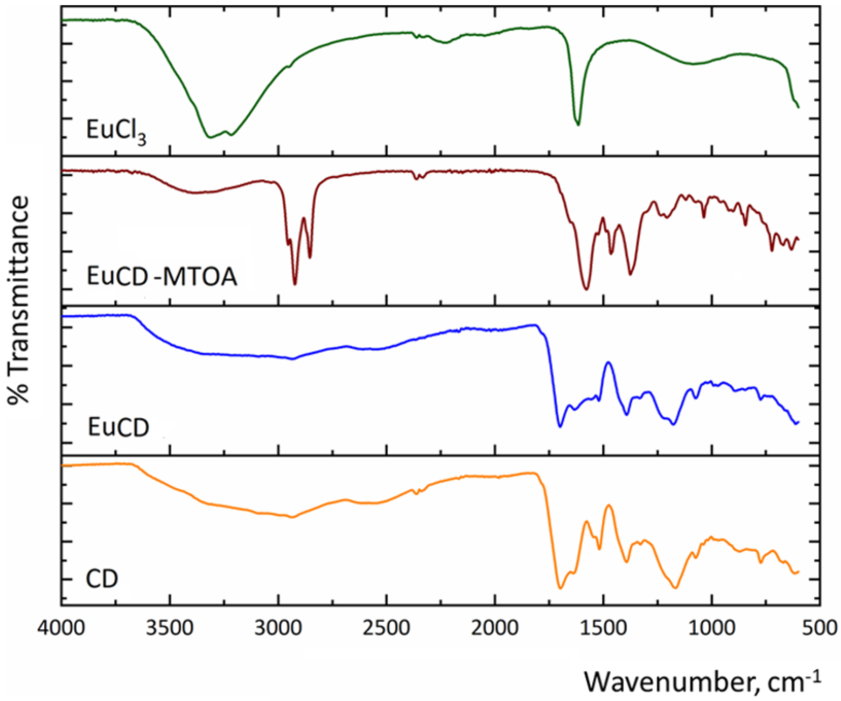

Figure SI.2 High resolution XPS spectra for C1s (left) and Eu3d5/2 (right)

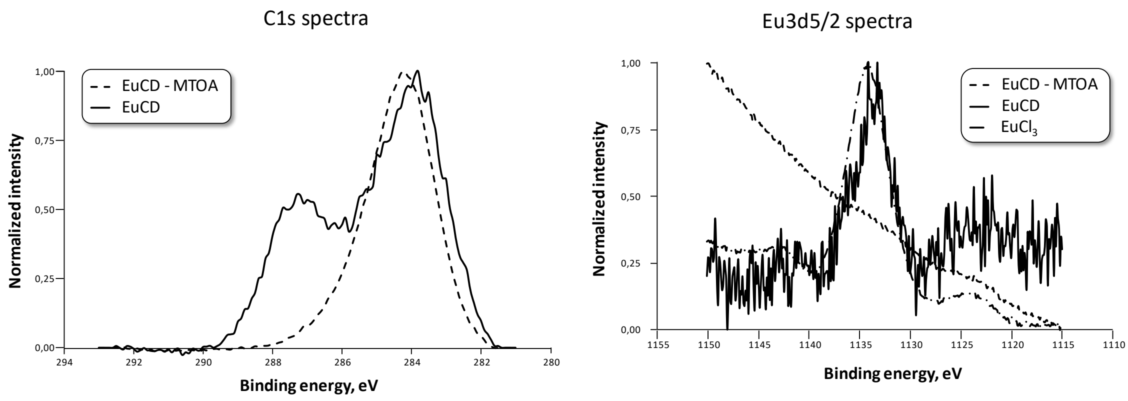

Figure SI.3. HRTEM images of (a) EuCD-MTOA; (b) structure detail of EuCD-MTOA; (c) STEM-HAADF images of EuCD-MTOA; (d) Size distribution for EuCD (mean 2.3 nm, SD 0.7 nm); (e) Size distribution for EuCD-MTOA (mean 2.0 nm, SD 0.5 nm)

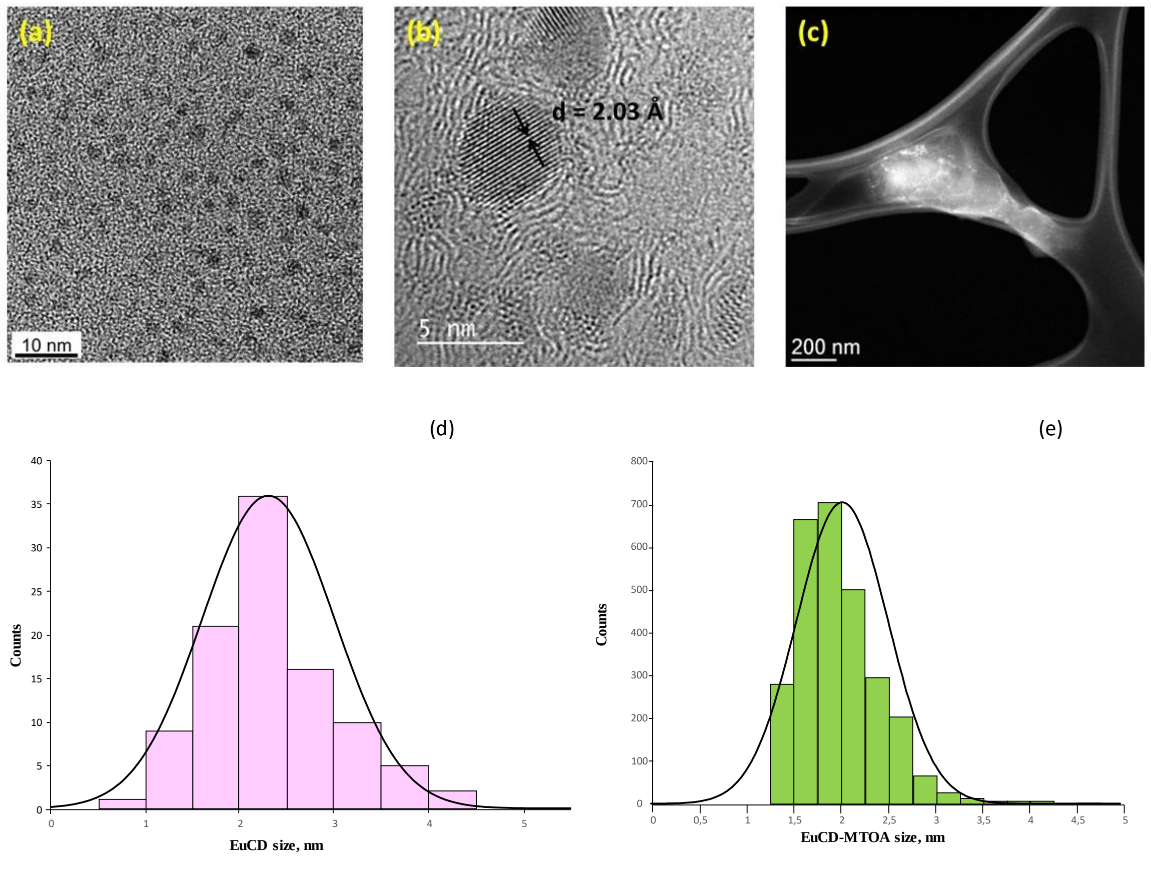

Figure SI.4. Emission spectra of the different carbon dots and europium salt in solid phase measured at Phosphorescence Standard Conditions (PSC: 0.1 ms delay time and 5 ms gate time).

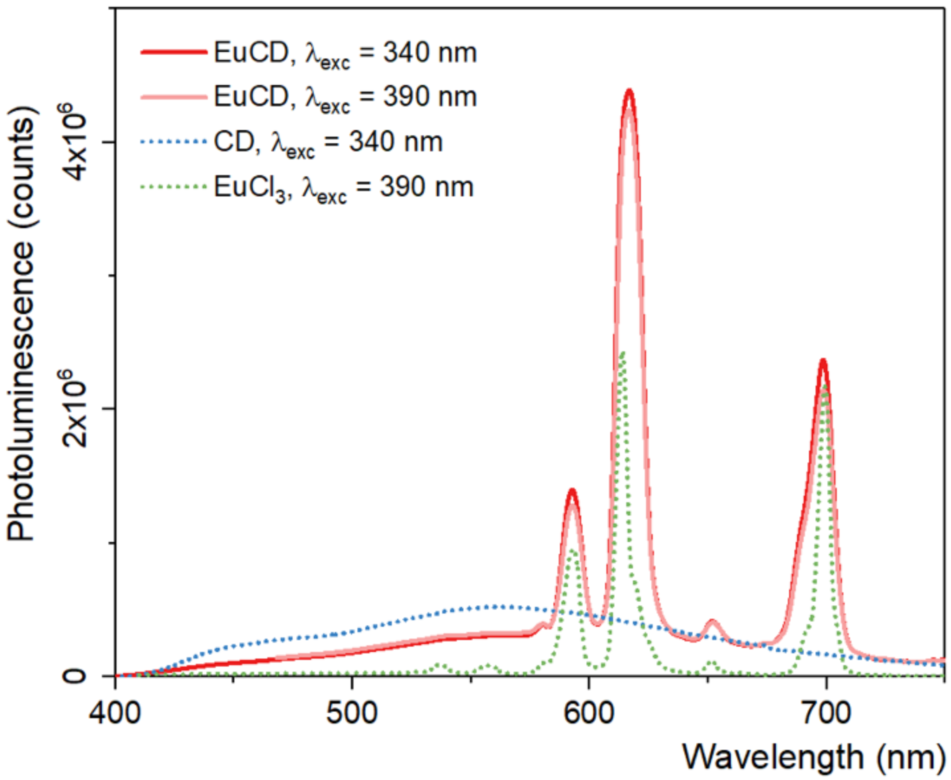

Figure SI.5. Photoluminescence emission of 0.1 % w/v EuCD-MTOA in organic solvents (PSC: 0.1 ms delay time and 5 ms gate time) and inset plot of $(Ph\nu)^2$ vs. photon energy (hν).

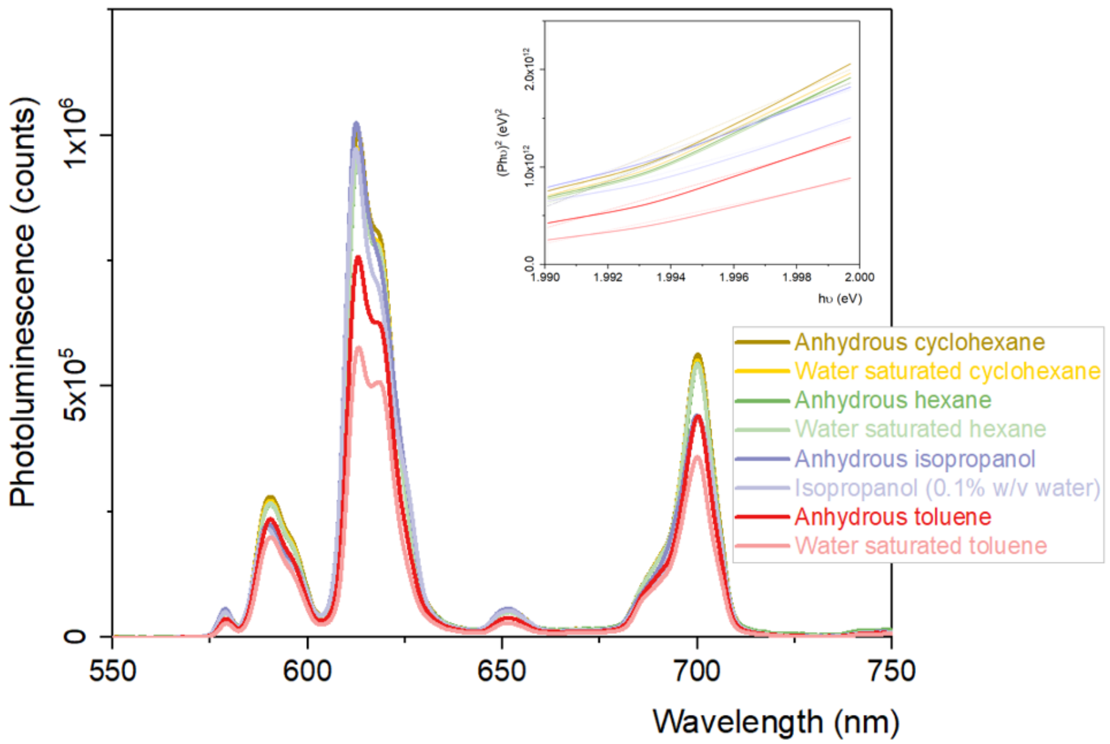

Figure SI.6. Evolution of the photoluminescence signal of the EuCD-MTOA dispersion in toluene over time. (a) The intensity was recorded every 60 minutes and (b) the intensity was recorded after two months.

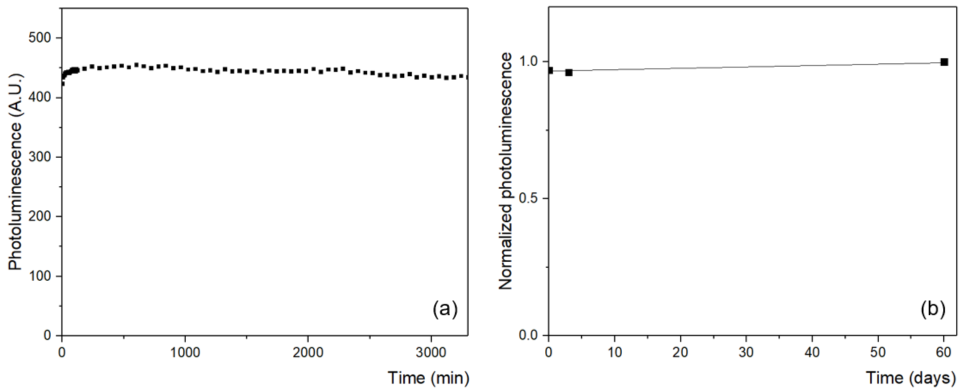

8. SI Tables

Table SI.1. Photoluminescence emission of EuCD-MTOA in 0.1 % w/v suspensions in organic solvents

Solvent	**Polarity index**	**Solvent KAT parameters**	**Normalized emission (▲)**	**Water solubility**

		α	β	π*		
Cyclohexane	0.04	0	0	0	100	0.021%
n-hexane	0.10	0	0	-0.08	95	0.022%
toluene	2.4	0	0.12	0.54	75	0.0245%
isopropanol	3.9	0.76	0.95	0.48	100	miscible

(▲) λ_{em}^{max}: 340 nm y λ_{exc}^{max}: 613 nm

Table SI.2. E_g^{613} experimental values obtained for EuCD-MTOA in different solvents with and without water.

Solvent	E_g^{613}	SD
Cyclohexane	2.0	0.2
Cyclohexane saturated	2.0	0.2
Hexane	2.0	0.2
Hexane saturated	2.0	0.2
Isopropanol	2.0	0.2
Dry isopropanol	2.0	0.1
Toluene	2.0	0.2
Toluene saturated	2.0	0.2

Table SI 3. Stern-Volmer fitted curves and analytical sensitivities for each carbon dot studied concentration (95% confidence intervals for parameters are included)

% w/v EuCD-MTOA in toluene	**Stern-Volmer fitted curve**	**Analytical sensitivity, γ (M^{-1})**
0.1	$P_0/P = (25 \pm 2)\,[H_2O] + (0.99 \pm 0.01)$	3862
0.05	$P_0/P = (21 \pm 3)\,[H_2O] + (1.00 \pm 0.02)$	3452
0.01	$P_0/P = (22 \pm 4)\,[H_2O] + (1.00 \pm 0.03)$	1545

Table SI.4. Relevant characteristics of developed methods to determine moisture present in non-polar solvents.

**Solvents**	**Nanomaterial**	**LOD**	**LOD / M**	**Ref.**
Toluene	CDs-naphthalimide-based PET sensor	1.2 ppm	$6.7\cdot10^{-5}$	38
n-hexane, dichloromethane, and toluene	Cs_4PbBr_6 NCs	0.031, 0.043, 0.057 µL mL^{-1}	$1,7\cdot10^{-3}$, $2.4\cdot10^{-3}$, $3.2\cdot10^{-3}$	39

EtOH, ACN, THF, and DiOX	CDs using 2,5-dihydroxyterephthalic acid	0.052, 0.0846, 0.103, and 0.176%	$2.9 \cdot 10^{-2}$, $4.7 \cdot 10^{-2}$, $5.7 \cdot 10^{-2}$& $9.8 \cdot 10^{-2}$	40
THF, acetone, DMSO, and methanol	hydroxy naphthaldehyde-based piezochromic luminogen, namely NAP-1	0.033, 0.032, 0.034, and 0.033%	$1.8 \cdot 10^{-2}$, $1.8 \cdot 10^{-2}$, $1.9 \cdot 10^{-2}$& $1.8 \cdot 10^{-2}$	41
Acetone	Microgels encapsulating AgNPS-DMAB-FPF	0.001%	$5.6 \cdot 10^{-4}$	42
Acetone, Isopropanol, THF	Karl Fisher Method	0.026, 0.054, 0.016%	$1.4 \cdot 10^{-2}$, $3.0 \cdot 10^{-2}$& $8.9 \cdot 10^{-3}$	43
Toluene	EuCD-MTOA	8.5×10^{-4} M	8.5×10^{-4}	This work
